# Brazil-Portugal Comparison: Education, Health and Social Development in light of the Sustainable Development Goals

**DOI:** 10.1590/0034-7167-2024-0047

**Published:** 2025-01-10

**Authors:** Felipa Rafaela Amadigi, Monica Motta Lino, Rosani Ramos Machado, Ianka Cristina Celuppi, Carla Sílvia Fernandes, Maria Manuela Martins, Denise Elvira Pires de Pires

**Affiliations:** I Universidade Federal de Santa Catarina. Florianópolis, Santa Catarina, Brazil; II Escola Superior de Enfermagem do Porto. Porto, Portugal; III Universidade do Porto. Porto, Portugal

**Keywords:** Sustainable Development, Diversity, Equity, Inclusion, Social Planning, Public Policy, Heath, Desarrollo Sostenible, Diversidad, Equidad e Inclusión, Planificación Social, Política Pública, Salud

## Abstract

**Objective:**

to comparatively analyze the health, education and social development systems of Brazil and Portugal, their relationship with the Sustainable Development Goals and the Organization for Economic Cooperation and Development averages.

**Method:**

exploratory and descriptive qualitative research, through documentary analysis. The indicators address health, education and social development, considering life expectancy, mortality, prevalence of chronic diseases, literacy, educational performance and poverty rates.

**Results:**

indicate significant differences between countries. Portugal presents better indicators in life expectancy, educational quality and poverty rates, whereas Brazil faces greater challenges in chronic diseases and equity in access to healthcare services.

**Final considerations:**

the importance of public policies adapted to local realities and the need for a strategic vision for healthcare systems aligned with the Sustainable Development Goals, in addition to the need for continuous investments and integration of digital health for efficient and equitable systems, stand out.

## RESUMEN

**Objetivo:**

analizar comparativamente los sistemas de salud, educación y desarrollo social de Brasil y Portugal, su relación con los Objetivos de Desarrollo Sostenible y los promedios de la Organización para la Cooperación y el Desarrollo Económico.

**Método:**

investigación cualitativa exploratoria y descriptiva, mediante análisis documental. Los indicadores abordan la salud, la educación y el desarrollo social, considerando la esperanza de vida, la mortalidad, la prevalencia de enfermedades crónicas, la alfabetización, el desempeño educativo y las tasas de pobreza.

**Resultados:**

indican diferencias significativas entre países. Portugal presenta mejores indicadores en esperanza de vida, calidad educativa y tasas de pobreza, mientras que Brasil enfrenta mayores desafíos en enfermedades crónicas y equidad en el acceso a los servicios de salud.

**Consideraciones finales:**

se destaca la importancia de políticas públicas adaptadas a las realidades locales y la necesidad de una visión estratégica de los sistemas de salud alineadas con los Objetivos de Desarrollo Sostenible, además de la necesidad de inversiones continuas e integración de la salud digital para sistemas eficientes y equitativos.

## INTRODUCTION

Public health and healthcare systems play a vital role in global socioeconomic development. According to the Organization for Economic Cooperation and Development (OECD) report, “Health at a Glance 2023”, Brazil and Portugal face several challenges, including chronic disease management and the adoption of digital health technologies. A comparative analysis of public data from both countries helps identify trends, common challenges and learning opportunities^(1)^. The intrinsic relationship between health and sustainability is evidenced by the understanding that health promotion is intertwined with sustainable practices.

The text analyzes the health, education and social development systems of Brazil and Portugal, exploring how these relate to the Sustainable Development Goals (SDGs) of the United Nations (UN) and the metrics established by the Organization for Economic Cooperation and Development (OECD). The study of these two countries, located on different continents, with different histories and economic realities, but which share Portuguese as their official language, is essential to understand the challenges and opportunities they face in promoting well-being of their populations. This analysis is particularly relevant in the current global scenario, in which understanding the variables that contribute to a dignified life becomes increasingly important^(1-5)^.

Among the similarities between the countries, the historical and cultural basis stands out, including the Portuguese language, despite being located on different continents. Both have predominantly public healthcare systems, which resemble the Beveridge model in terms of public financing and government administration^(6)^. They adopt decentralized healthcare systems, although Brazil has a lower population density compared to Portugal. Both countries share the principle that it is the State’s duty to guarantee the population’s health, but the tripartite federative organization in Brazil contrasts with the unitary system in Portugal. Furthermore, both conceive health from a comprehensive perspective as a fundamental right, recognizing the importance of considering diverse factors to promote well-being.

The social determinants of health, as defined by the World Health Organization, encompass socioeconomic, environmental and cultural conditions that shape well-being. They include aspects such as living conditions, work, access to education and healthcare services, reflecting individuals’ life cycle. On the other hand, the health-disease process social determination, which originated in Latin America, expands this view to address broader social, economic and political influences. This approach highlights the need for interdisciplinary and practices collaboration between sectors such as health, education and justice to address the social roots of health inequalities. Challenging traditional paradigms, this perspective sees health not only as a result of individual choices, but as intrinsically linked to social conditions that shape people’s lives^(7,8)^.

From this perspective, recognizing the interconnection between these determinants and health is crucial to understanding existing disparities and developing effective strategies to promote overall well-being. Equitable access to the social determinants of health is essential to ensuring that everyone has the opportunity to achieve their full health potential. Inequalities in areas such as education, income, and housing can result in significant disparities in health outcomes. Therefore, policies and interventions aimed at promoting health should address not only clinical aspects, but also the social conditions that shape people’s lives in society^(7-12)^.

An important agent for the exchange of experiences between nations is OECD, composed of 38 member countries, of which Portugal is a member. Brazil, although not a full member, is an active partner of OECD, collaborating on several initiatives and contributing to discussions on economic and social policies. On the one hand, OECD presents the analysis of trends, but, on the other, the UN proposes, through the 17 SDGs, interconnected objectives to address the socioeconomic and environmental challenges faced worldwide by 2030. These goals cover a range of issues, from eradication of poverty to promotion of gender equality, quality education, climate action, and peace and justice^(13)^.

Analyzing nations’ performance and its relationship to the SDGs provides a broader perspective on how countries are progressing toward global health goals.

## OBJECTIVE

To comparatively analyze the health, education and social development systems of Brazil and Portugal, their relationship with the SDGs and averages of the OECD averages.

## METHODS

### Ethical aspects

In this research, ethical principles that guarantee integrity, reliability and respect for human rights were strictly observed. The data used are public or from official sources. There are no conflicts of interest, and the research was conducted with impartiality and neutrality.

### Study design

This is exploratory and descriptive qualitative research, based on documentary analysis^(14,15)^ of several reliable and relevant data sources, detailed in the “Data sources” section.

### Methodological procedures

This comparative study between Brazil and Portugal analyzed health, education and social development indicators. Data were collected in January and February 2024. To compare indicators between Brazil and Portugal, OECD reports^(1,2)^ and data from the Program for International Student Assessment (PISA) were used. To obtain specific information about Brazil, data from the Institute of Applied Economic Research (IPEA - *Instituto de Pesquisa Econômica Aplicada*) and e-gestor, a website with indicators from the Brazilian government, were used.

Finally, a comparative synthesis of the results found for Brazil and Portugal was prepared, highlighting similarities, differences and trends observed in relation to OECD average. It is important to note that there are no conflicts of interest in this work.

### Study setting

The research originated from data available in the reports “Health at a Glance 2023”^(1)^ and “Education at a Glance 2023”^(2)^, published by OECD. Data presented in PISA results were subsequently added.

We then opted for an online research approach to investigate the health, education and social development systems in the chosen countries, due to ease of searching for official documents from government agencies and indexed academic journals. The search was restricted to documents published in the last five years to ensure that information was up-to-date.

### Data sources

To carry out a comparative analysis of the health, education and social development situation in Brazil and Portugal, several reliable and relevant data sources were used, including:

- OECD reports and studies, which provide indicators and data on health, education and social development in member countries, including Portugal;
- PISA data to assess the quality of education in both countries;
- Reports from non-governmental organizations and academic institutions that focus on sustainable development issues, such as IPEA in Brazil and the e-gestor system.

### Data collection and organization

In data collection, selected indicators cover the areas of health status, education and social development (Chart 1).

**Chart 1:** Characterization of indicators in the areas of health status, education and social development

Health status	Life expectancy at birth; preventable mortality rate; prevalence of chronic conditions such as diabetes; self-rated health by the population; infant mortality rate; maternal mortality rate; cause-specific mortality rate; daily smoking rate; alcohol consumption per capita; prevalence of obesity; population coverage of healthcare services; population satisfaction with the availability of healthcare.
Education	Literacy rate; secondary school completion rate; PISA performance in mathematics; PISA performance in reading; PISA performance in sciences.
Social development	Poverty rate; income inequality; unemployment rate; social inclusion rate.

*PISA - Programa Internacional de Avaliação de Alunos.*

The data were organized according to health status, education and social development in Brazil and Portugal.

### Data analysis

The data were analyzed comparatively between Brazil and Portugal. In health status, the following were analyzed: Comparison of maternal and infant mortality by cause; Comparison of life expectancy and preventable mortality rate; Comparison of prevalence of chronic diseases; Self-rated health; Risk factors for health; and Comparison of healthcare service coverage. In education, the following were analyzed: Comparison of literacy and school completion rates; Comparison of investment in education; and PISA results. In social development, the following were analyzed: Comparison of poverty rates; Comparison of income inequality; Unemployment rate; Social inclusion rate. Finally, a comparative synthesis was prepared between Brazil and Portugal and OECD average. Statistical methods, such as mean, median and standard deviation, were used, when appropriate, to identify significant differences between countries.

## RESULTS

The results are organized according to health status, education and social development in Brazil and Portugal.

### Health status

#### Comparison of maternal and infant mortality by cause

Infant mortality and maternal mortality are important indicators of the quality and effectiveness of healthcare systems as well as the level of social vulnerability of people. In this regard, Portugal has better results than Brazil and OECD average^(16,17)^.

Portugal also has better results in mortality rates from causes related to the circulatory and respiratory systems, totaling 222 (per 100,000 people) and 82 (per 100,000 people), respectively, whereas Brazil achieved values of 340 (per 100,000 people) in mortality related to the circulatory system and 152 (per 100,000 people) in mortality related to the respiratory system. Portugal has a worse performance in mortality related to neoplasms, totaling 211 (per 100,000 people), whereas Brazil registered 178 (per 100,000 people), rate below the OECD average^(16)^.

#### Comparison of life expectancy and preventable mortality rate

Life expectancy at birth is a key indicator for assessing quality of life and access to healthcare. Portugal stands out positively in this regard, with a life expectancy significantly higher than OECD average. In 2021-2022, life expectancy in Portugal was 81.5 years, whereas OECD average was 80.3 years. In Brazil, life expectancy was 74.0 years, still below the OECD average^(16)^.

The preventable mortality rate reflects the effectiveness of healthcare systems in preventing deaths that could be avoided with adequate healthcare. Portugal has a lower preventable mortality rate than OECD average, suggesting a more efficient healthcare system. However, Brazil faces challenges in this regard, with a higher preventable mortality rate than OECD average^(16)^.

#### Comparison of prevalence of chronic diseases

Prevalence of chronic diseases is another important health indicator. In Brazil, 8.8% of the adult population is affected by diabetes, whereas in Portugal this figure is 9.1%. Both figures are above the OECD average of 7.0%. This indicates the need for more effective prevention and control policies in both countries^(18)^.

The proportion of underreporting of diabetes mellitus in Brazil was 42.5%, reaching 72.8% in the North ^(19)^. In Portugal, considering the composition of the prevalence rate of diabetes, in 56% of individuals, it had already been diagnosed, and in 44%, it had not yet been diagnosed^(20)^.

#### Self-rated health

Self-rated health is a subjective indicator in which individuals self-rate their own health. In Portugal, 77.3% of the population aged 15 or over classify their health as good or very good, whereas in Brazil this figure is 65.7%. Both countries are below the OECD average of 70.2%. This subjective assessment can be influenced by several factors, including access to healthcare and quality of life^(1)^.

#### Risk factors for health

Analyzing risk factors for health, we see that Portugal has a higher percentage of daily smokers compared to Brazil. Thus, 14.2% of the Portuguese population aged 15 or over are daily smokers, whereas in Brazil this number is 9.1%. OECD average is 15.9%. As for alcohol consumption, Portugal has a higher rate, with 10.4 liters consumed per capita over the age of 15, compared to 9.8 liters in Brazil, and OECD average of 8.6 liters. Obesity affects 17% of the population aged 15 or over in Portugal and 22% in Brazil, both above the OECD average of 18%^(16)^.

#### Comparison of healthcare service coverage

Regarding healthcare service coverage, Portugal has a high percentage of the population covered by the basic set of services, with 95.1% of the population benefiting from this access. In Brazil, this number is 76.08%. Portugal is above the OECD average, which is 80.2%, whereas Brazil is below. Population satisfaction with the availability of quality healthcare is also higher in Portugal, with 73.8% of the population satisfied, whereas in Brazil this number is 57.4%. OECD average for population satisfaction is 70.8%^(1,21)^.

### Education

#### Comparison of literacy and school completion rates

In the education dimension, we begin by comparing literacy and school completion rates. Portugal has a higher literacy rate than Brazil and OECD average. Furthermore, 96.3% of the population in Portugal is literate, whereas in Brazil this number is 93.2%. OECD average is 98.1%. In terms of school completion, Portugal also stands out, with 52.7% of the population completing secondary education, whereas in Brazil this number is 47.9%. OECD average is 84.3%^(22)^.

#### Comparison of investment in education

Investment in education is essential to ensure the quality and accessibility of the education system. In Portugal, the investment per student is US$9,100.00 per year, whereas in Brazil, this figure is US$3,600.00 per year. Both values are below the OECD average, which is US$11,200.00. This disparity in investment may explain, in part, the differences observed in the quality of education between the two countries^(2)^.

#### Resulsts from the Programme for International Student Assessment

PISA assesses student performance in several areas, including mathematics, reading and science. Portugal has achieved results above the OECD average in some areas of PISA, whereas Brazil is below the average. This suggests a higher quality of Portuguese education compared to Brazil.

Brazil invested, from primary to higher education in 2020, US$4,306.00 per student, equivalent to approximately R$ 21,500.00, whereas OECD countries invested, on average, US$11,560.00, or R$ 57,800.00, according to an OECD report^(2)^. Consequently, it presents unsatisfactory academic results in international assessments. In PISA, Brazil has a below-average performance in the groups of mathematics (379, -5 compared to 2018), reading (410, -3 compared to 2018) and sciences (403, -1 compared to 2018). Portugal obtained a performance of 472 points in mathematics (-20.6 compared to 2018), 477 in reading (-15.2 compared to 2018) and 485 in sciences (-7.3 compared to 2018), with OECD average being, respectively, 472, 477 and 485, placing it in the group of countries not statistically different from OECD average^(23)^. Portugal, in turn, invested US$12,104.00 per student in education in 2020^(22)^.

Overall, PISA 2022 results have declined due to the impact of COVID-19. OECD average dropped by almost 14 points in mathematical literacy and around 10 in reading, compared to PISA 2018. However, in the case of Brazil, school closures do not show a significant difference in results^(24)^.

### Social development

#### Comparison of poverty rates

In the social development dimension, the comparison of poverty rates reveals significant differences between Portugal and Brazil. Portugal has a lower poverty rate than Brazil and is close to OECD average. In Portugal, 13.4% of the population lives below the poverty line, whereas in Brazil this number is 21.4%. OECD average is 11.7%^(25)^.

#### Comparison of income inequality

Income inequality, as measured by the Gini coefficient, is lower in Portugal than in Brazil. Portugal is close to OECD average in terms of income inequality, whereas Brazil has significantly higher income inequality than OECD average^(26)^.

#### Unemployment rate

Unemployment rates also differ between the two countries. Portugal has a lower unemployment rate compared to Brazil. In Portugal, 6.6% of the population is unemployed, whereas in Brazil, this number is 14%^(26)^.

#### Social inclusion rate

Portugal and Brazil face challenges in terms of social inclusion. Both countries have social inclusion rates below the OECD average. In Portugal, 17.2% of the population faces social exclusion, whereas in Brazil this figure is 24.8%. OECD average is 14.3%^(25-26)^.

### Comparative synthesis between Brazil and Portugal and Organization for Economic Cooperation and Development average

Table 1 shows the comparative synthesis of indicators of Brazil and Portugal with OECD average.

**Table 1:** Indicators for Brazil and Portugal and Organization for Economic Cooperation and Development average

	Year	Brazil	Portugal	OECD average
Health status				
Life expectancy (years)	2022	74.0	81.5	80.3
Avoidable mortality (per 100,000 people)	2021	257	114	158
Prevalence of diabetes (%)	2021	8.8%	9.1%	7.0%
Infant mortality (per 100,000 people)	2021	12.5	2.4	4.0
Maternal mortality (per 100,000 people)	2020	72.2	11.8	10.9
Mortality rate from causes related to the circulatory system (per 100,000 people)	2021	340	222	286
Mortality rate due to causes related to neoplasms (per 100,000 people)	2021	178	211	202
Mortality rate due to causes related to the respiratory system (per 100,000 people)	2021	152	82	67
Coverage of essential services - Primary Care (%)	2020	76.08	95.10	80.20
Population >15 years who smoke daily (%)	2021	9.1	14.2	15.9
Population >15 years who consume alcohol daily (%)	2021	9.8	10.4	8.6
Self-reported rates of overweight and obesity among adults (%)	2021	22	17	18
Self-rated health	2021	65.7	77.3	70.2
Education				
Literacy rate (%)	2022	93.2%	96.3%	98.1%
Secondary school completion rate (%)	2022	47.9%	52.7%	84.3%
PISA performance in mathematics (points)	2022	379	472	472
PISA performance in reading (points)	2022	410	477	477
PISA performance in sciences (points)	2022	403	485	485
Social development				
Poverty rate (%)	2022	21.4%	13.4%	11.7%
Income inequality (points)	2020	48.9	34.7	33.13
Unemployment rate (%)	2021	14%	6.6%	6.1
Social exclusion rate (%)	2022	24.8%	17.2%	14.3%

*Source: PISA (2022); OECD (2023); e-gestor (2021).*

*OECD - Organization for Economic Cooperation and Development; PISA - Programme for International Student Assessment*

## DISCUSSION

The results reveal significant differences between the health, education and social development situations in Brazil and Portugal as well as in comparison with OECD average. The continental size of Brazil, in comparison with Portugal, may be one of the factors that hinder the implementation of more assertive and equitable public policies. We will now discuss these results in light of the UN SDGs, exploring how these indicators relate to the SDGs and the implications for public policies.

### Health and Sustainable Development Goal 3 - Good Health and Well-Being

UN SDG 3 aims to ensure healthy lives and promote well-being for all at all ages. An analysis of health indicators in both countries shows that Portugal performs better in relation to this goal than Brazil.

Portugal surpasses Brazil in life expectancy, preventable mortality rate and self-rated health. This suggests that health policies in Portugal have been more effective in promoting health and preventing disease. However, both countries face challenges related to chronic diseases and equity in access to healthcare services. The aging of the Portuguese population poses specific challenges, especially in health, which requires an integrated approach in terms of public policies^(27)^.

The literature highlights three priorities for the Portuguese healthcare system, which are improving quality of access, motivating healthcare professionals to increase their productivity and providing financial resources to cover costs and investment of health actions^(28)^.

The healthcare system in Brazil stands out positively for offering universal coverage. However, it faces challenges with the quality and efficiency of services, in addition to significant regional disparities. The Portuguese healthcare system, on the other hand, is well assessed in terms of access and quality. For Brazil, achieving SDG 3 will require a significant increase in investments in health, improvement in the quality of services and promotion of disease prevention strategies. The digitalization of health can be a catalyst for transformations in this sense, improving access and efficiency of healthcare services, since access to health encompasses the presence of resources and services, as well as acceptability, which acts as a driver for choosing these services, as it facilitates achieving goals, i.e., the effective response to needs, encompassing ease of access^(11,29-31)^.

Aligning SDG goals and targets with the specific demands of the Brazilian Healthcare system (SUS - *Sistema Único de Saúde*) is essential to ensure effective compliance with SDGs, whereas simultaneously strengthening the operationality of SUS. By incorporating SDG goals into the planning and implementation of health policies, a viable path to achieving universalization and equity in health is created^(4)^.

### Education and Sustainable Development Goal 4 - Quality Education

Since 2000, the world has made significant progress in education, marked by the establishment of the six Education For All goals and the Millennium Development Goals. Despite efforts, these goals were not achieved within the 2015 deadline, highlighting the need for continued commitment to completing the unfinished agenda and, therefore, here we are with the 2030 Agenda for Sustainable Development “Ensure inclusive and equitable quality education and promote lifelong learning opportunities for all”^(5)^.

UN SDG 4 seeks to ensure inclusive, equitable and quality education, promoting lifelong learning opportunities for all. An analysis of educational indicators reveals that Portugal is more aligned with this goal than Brazil.

Recognizing the relevance of education, the 2030 Agenda for Sustainable Development highlights education as a stand-alone goal (SDG 4), whereas also incorporating education targets into several other SDGs, especially those related to health, economic growth, employment, sustainable consumption and production, and climate change. Education, in fact, has the potential to drive progress towards the achievement of all SDGs and should therefore be integrated into strategies to achieve them. The new approach to education outlined in SDG 4 is comprehensive, holistic, ambitious and global, guided by a transformative vision of education that positively impacts individuals’, communities’ and societies’ lives, ensuring that no one is left behind^(5)^.

Portugal surpasses Brazil in literacy rates, school completion and PISA performance. This shows that education policies in Portugal have been more effective in promoting quality and accessible education. Investment in education and the reforms implemented have been fundamental to this progress.

In the case of Brazil, achieving SDG 4 will require a significant increase in investment in education, as well as efforts to improve the quality of education and reduce educational inequalities. Expanding access to higher education must also be accompanied by the promotion of real employment opportunities, in line with SDG 8.

For change to be possible and for this goal to be achieved, governments must take primary responsibility for guaranteeing the right to education, playing a central role as guardians of the efficient, equitable and effective management and financing of public education and maintaining political leadership in the education sector. They must guide the process of contextualizing and implementing the objectives and goals established by education, but it is up to society to remain remain vigilant^(5,31)^.

### Social development and Sustainable Development Goal 1 - No Poverty and Sustainable Development Goal 10 - Reduced Inequalities

UN SDGs 1 and 10 call for eradication of poverty and reduced inequalities. Analysis of social development indicators reveals significant differences between Brazil and Portugal.

Portugal has a lower poverty rate and lower income inequality than Brazil. This suggests that social policies in Portugal have been more effective in promoting social inclusion and reducing economic disparities. However, both countries face challenges in terms of social inclusion, with rates below the OECD average.

Brazil’s striking inequality, aligned with SDG 10, calls into question not only income redistribution policies, but the social structure itself. How can interventions go beyond mitigation to eliminate the structural roots of inequalities? Portugal, facing challenges even with smaller inequalities, raises questions about the nature of these inequalities. How can policies be adapted to address specific challenges, whereas respecting the uniqueness of the Portuguese context?

In Brazil, achieving SDGs 1 and 10 will require implementing public policies aimed at reducing poverty and inequality, progressively achieving and sustaining income growth for the poorest 40% of the population at a rate higher than the mean income of the richest 10% as well as promoting economic opportunities for all. Social inclusion must be prioritized, with actions that serve the most vulnerable groups^(10-12,30,31)^.

Addressing the problems identified is strongly dependent on macro-policies that have continuity and identity, with values of equality, solidarity and human respect.

### Comparison with the Organization for Economic Cooperation and Development average and ethical aspects

Comparison with OECD average shows that both Portugal and Brazil have areas that are below this international standard. This highlights the complexity of the challenges faced by both countries and the need for action to improve their health, education and social development systems.

Furthermore, it is essential to consider the ethical aspects involved when analyzing indicators related to health, education and social development. Public policies must be guided by ethical principles that promote equal access, social justice and respect for human rights. Data collection and use must also be conducted ethically, ensuring the privacy and confidentiality of citizens’ information.

### Study limitations

Finally, some limitations of this study include differences in scale and population, historical and cultural context, internal diversity, distinct political and administrative systems, social and economic inequalities, and the definition and measurement of indicators.

### Contributions to nursing, health or public policy

This study enriches the debate on public health policies, highlighting the importance of a multifaceted approach that considers not only health indicators, but also educational and social development factors. The findings point to the need for policies that promote equity and efficiency, encouraging Brazil and Portugal to inspire each other in the areas where each excels. By highlighting the central role of health in sustainable development, the study invites policymakers to consider long-term strategic investments that address not only immediate needs, but also lay the foundation for healthier and more resilient societies.

## FINAL CONSIDERATIONS

The health, education and social development indicators of Brazil and Portugal, analyzed and compared with OECD average, provide an insight into the disparities between the two countries. Portugal stands out positively in several areas, including life expectancy, quality of education and poverty rates. However, both countries face challenges, such as the high prevalence of chronic diseases and the need for investment in education.

The comparative analysis shows that both countries have aspects in which they align with or diverge from OECD average, with Portugal generally being closer to OECD standards than Brazil. The analysis also highlights the complexity and variety of challenges faced by Brazil and Portugal, illustrating how historical, economic and political factors influence performance in health, education and social development. The trends indicate areas of progress and opportunities for future reforms and investments that can guide public policies in the fields of health, education and social development.

By highlighting the differences and similarities between Brazil and Portugal in relation to OECD averages in health, education and social development, this study offers a comprehensive overview of the challenges to be faced by both countries as well as areas of emphasis that can serve as a model for other nations.

It is imperative that both Brazil and Portugal maintain a continued commitment to improving their healthcare systems. A special emphasis on digital health integration and equitable distribution of resources is a pressing need. Furthermore, the adoption of policies aligned with the UN SDGs is crucial to ensure consistent progress towards more efficient and equitable healthcare systems.

The challenge for the future lies in the persistence of investments, recognizing that health is a fundamental pillar for sustainable development. The path towards more robust healthcare systems requires not only the resolution of immediate challenges, but also a strategic vision that anticipates and responds promptly to future demands of society.
