# Supplementary material for: Brazil-Portugal Comparison: Education, Health and Social Development in light of the Sustainable Development Goals
Source: Rev Bras Enferm. 2025 Jan 10;77(Suppl 2):e20240047. doi: 10.1590/0034-7167-2024-0047 (PMC11726810; doi:10.1590/0034-7167-2024-0047)
Supplement: Translation

# Translation: Comparação Brasil-Portugal: Educação, Saúde e Desenvolvimento Social à luz dos Objetivos de Desenvolvimento Sustentável

**DOI:** 10.1590/0034-7167-2024-0047pt

**Authors:** Felipa Rafaela Amadigi, Monica Motta Lino, Rosani Ramos Machado, Ianka Cristina Celuppi, Carla Sílvia Fernandes, Maria Manuela Martins, Denise Elvira Pires de Pires

**Affiliations:** I Universidade Federal de Santa Catarina. Florianópolis, Santa Catarina, Brasil; II Escola Superior de Enfermagem do Porto. Porto, Portugal; III Universidade do Porto. Porto, Portugal

**Keywords:** Desenvolvimento Sustentável, Diversidade, Equidade, Inclusão, Planejamento Social, Política Pública, Saúde

## Abstract

**Objetivo:**

analisar comparativamente o sistema de saúde, educação e desenvolvimento social de Brasil e Portugal, sua relação com os Objetivos de Desenvolvimento Sustentável e as médias da Organização para Cooperação e Desenvolvimento Econômico.

**Método:**

pesquisa qualitativa exploratória e descritiva, através de análise documental. Os indicadores abordam saúde, educação e desenvolvimento social, considerando expectativa de vida, mortalidade, prevalência de doenças crônicas, alfabetização, desempenho educacional e taxas de pobreza.

**Resultados:**

indicam diferenças significativas entre os países. Portugal apresenta melhores indicadores em expectativa de vida, qualidade educacional e taxas de pobreza, enquanto que Brasil enfrenta maiores desafios em doenças crônicas e equidade no acesso aos serviços de saúde.

**Considerações finais:**

ressaltam-se a importância de políticas públicas adaptadas às realidades locais e a necessidade de uma visão estratégica para sistemas de saúde alinhados aos Objetivos de Desenvolvimento Sustentável, além da necessidade de investimentos contínuos e integração da saúde digital para sistemas eficientes e equitativos.

## INTRODUÇÃO

A saúde pública e os sistemas de saúde desempenham um papel vital no desenvolvimento socioeconômico global. De acordo com o relatório “*Health at a Glance 2023*” da Organização para Cooperação e Desenvolvimento Econômico (OCDE), Brasil e Portugal enfrentam diversos desafios, incluindo o gerenciamento de doenças crônicas e a adoção de tecnologias de saúde digital. Uma análise comparada de dados públicos dos dois países contribui para identificar tendências, desafios comuns e oportunidades de aprendizado^(1)^. A relação intrínseca entre saúde e sustentabilidade é evidenciada pela compreensão de que a promoção da saúde está entrelaçada com práticas sustentáveis.

O texto em tela analisa os sistemas de saúde, educação e desenvolvimento social de Brasil e de Portugal, explorando como esses se relacionam com os Objetivos de Desenvolvimento Sustentável (ODS) da Organização das Nações Unidas (ONU) e com as métricas estabelecidas pela Organização para a Cooperação e Desenvolvimento Econômico (OCDE). O estudo desses dois países, localizados em continentes distintos, com diferentes históricos e realidades econômicas, mas que compartilham a língua portuguesa como idioma oficial, é essencial para compreender os desafios e as oportunidades que eles enfrentam na promoção do bem-estar de suas populações. Esta análise é particularmente relevante no atual cenário global, em que entender as variáveis que contribuem para uma vida digna se torna cada vez mais importante^(1-5)^.

Entre as semelhanças dos países, destaca-se a base histórica e cultural, incluindo a língua portuguesa, apesar de estarem localizados em continentes distintos. Ambos possuem sistemas de saúde predominantemente públicos, que se assemelham ao modelo Beveridge em termos de financiamento público e administração governamental^(6)^. Adotam sistemas de saúde descentralizados, embora o Brasil possua menor densidade populacional em comparação com Portugal. Os dois países compartilham o princípio de que é dever do Estado garantir a saúde da população, mas a organização federativa tripartite no Brasil contrasta com o sistema unitarista de Portugal. Além disso, ambos concebem a saúde sob uma perspectiva integral como direito fundamental, reconhecendo a importância de considerar fatores diversos para promover o bem-estar.

Os determinantes sociais da saúde, segundo a definição da Organização Mundial da Saúde, englobam condições socioeconômicas, ambientais e culturais que moldam o bem-estar. Incluem aspectos como condições de vida, trabalho, acesso à educação e serviços de saúde, refletindo o ciclo de vida das pessoas. Por outro lado, a determinação social do processo saúde-doença, originada na América Latina, amplia essa visão para abordar influências sociais, econômicas e políticas mais amplas. Essa abordagem destaca a necessidade de colaboração interdisciplinar entre setores como saúde, educação e justiça para enfrentar as raízes sociais das desigualdades em saúde. Desafiando paradigmas tradicionais, essa perspectiva enxerga a saúde não apenas como resultado de escolhas individuais, mas como intrinsecamente ligada às condições sociais que moldam a vida das pessoas^(7,8)^.

Partindo desta concepção, reconhecer a interconexão entre esses determinantes e a saúde é crucial para compreender as disparidades existentes e desenvolver estratégias eficazes para promover o bem-estar geral. O acesso equitativo aos determinantes sociais da saúde é essencial para garantir que todos tenham a oportunidade de atingir seu máximo potencial de saúde. A desigualdade em áreas como educação, renda e habitação pode resultar em disparidades significativas nos resultados de saúde. Portanto, políticas e intervenções voltadas para a promoção da saúde devem abordar não apenas os aspectos clínicos, mas também as condições sociais que moldam as vidas das pessoas em sociedade^(7-12)^.

Um agente importante para a troca de experiências entre nações é a OCDE, composta por 38 países membros, da qual Portugal faz parte. O Brasil, embora não seja um membro pleno, é um parceiro ativo da OCDE, colaborando em diversas iniciativas e contribuindo para discussões sobre políticas econômicas e sociais. De um lado, a OCDE apresenta a análise de tendências, mas, de outro, a ONU propõe, por meio dos 17 ODS, objetivos interconectados, abordar os desafios socioeconômicos e ambientais enfrentados em todo o mundo até 2030. Esses objetivos abrangem uma gama de questões, desde a erradicação da pobreza até a promoção da igualdade de gênero, educação de qualidade, ação climática, e paz e justiça^(13)^.

Analisar o desempenho das nações e a sua relação com os ODS fornece uma perspectiva mais ampla sobre como os países estão progredindo em direção a metas de saúde globais.

## OBJETIVO

Analisar comparativamente o sistema de saúde, educação e desenvolvimento social de Brasil e Portugal, sua relação com os ODS e as médias da OCDE.

## MÉTODOS

### Aspectos éticos

Nesta pesquisa, foram rigorosamente observados princípios éticos que garantem a integridade, confiabilidade e respeito pelos direitos humanos. Os dados utilizados são públicos ou de fontes oficiais. Não há conflitos de interesse, e a pesquisa foi conduzida com imparcialidade e neutralidade.

### Tipo de estudo

Pesquisa qualitativa exploratória e descritiva, a partir de análise documental^(14,15)^ de diversas fontes de dados confiáveis e relevantes, detalhadas na seção “Fontes de dados”.

### Procedimentos metodológicos

Este estudo comparativo entre Brasil e Portugal analisou os indicadores de saúde, educação e desenvolvimento social. Os dados foram coletados durante os meses de janeiro e fevereiro de 2024. Para comparar indicadores entre Brasil e Portugal, fez-se o uso de relatórios da OCDE^(1,2)^ e dados do Programa Internacional de Avaliação de Alunos (PISA). Para obter informações específicas sobre o Brasil, fez-se o uso de dados do Instituto de Pesquisa Econômica Aplicada (IPEA) e do e-gestor, site com indicadores do governo brasileiro.

Ao final, foi elaborada uma síntese comparativa dos resultados encontrados para Brasil e Portugal, destacando semelhanças, diferenças e tendências observadas em relação à média da OCDE. Destaca-se que neste trabalho não há conflitos de interesse.

### Cenário do estudo

A pesquisa se originou a partir dos dados disponíveis nos relatórios “*Health at a Glance 2023*”^(1)^ e “*Education at a Glance 2023*”^(2)^, publicados pela OCDE. Posteriormente, agregaram-se dados apresentados nos resultados do PISA.

Em seguida, optamos por uma abordagem de pesquisa *online* para investigar os sistemas de saúde, educação e desenvolvimento social nos países escolhidos, devido à facilidade na busca de documentos oficiais de órgãos governamentais e de periódicos acadêmicos indexados. A busca foi restrita a documentos publicados nos últimos cinco anos para garantir a atualidade das informações.

### Fontes de dados

Para realizar uma análise comparada da situação de saúde, educação e desenvolvimento social no Brasil e em Portugal, foram utilizadas diversas fontes de dados confiáveis e relevantes, incluindo:

Relatórios e publicações da OCDE, que fornecem indicadores e dados sobre saúde, educação e desenvolvimento social em países membros, incluindo Portugal;

Dados do PISA para avaliar a qualidade da educação em ambos os países;

Relatórios de organizações não governamentais e instituições acadêmicas que se concentram em questões de desenvolvimento sustentável, como o IPEA no Brasil e o sistema e-gestor.

### Coleta e organização dos dados

Na coleta de dados, os indicadores selecionados abrangem as áreas de estado de saúde, educação e desenvolvimento social (Quadro 1).

**Quadro 1:** Caracterização dos indicadores das áreas de estado de saúde, educação e desenvolvimento social

Estado de saúde	Expectativa de vida ao nascer; taxa de mortalidade evitável; prevalência de condições crônicas, como diabetes; autoavaliação de saúde pela população; taxa de mortalidade infantil; taxa de mortalidade materna; taxa de mortalidade por causa específica; taxa de fumantes diários; consumo de álcool *per capita*; prevalência de obesidade; cobertura populacional de serviços de saúde; satisfação da população com a disponibilidade de cuidados de saúde.
Educação	Taxa de alfabetização; taxa de conclusão escolar do ensino secundário; desempenho no PISA na categoria matemática; desempenho no PISA na categoria leitura; desempenho no PISA na categoria ciências.
Desenvolvimento social	Taxa de pobreza; desigualdade de renda; taxa de desemprego; taxa de inclusão social.

*PISA - Programa Internacional de Avaliação de Alunos.*

Os dados foram organizados conforme o estado de saúde, educação e desenvolvimento social no Brasil e em Portugal.

### Análise dos dados

Os dados foram analisados de forma comparativa entre Brasil e Portugal. No estado de saúde, foram analisados: Comparação da mortalidade por causa, materna e infantil; Comparação da expectativa de vida e taxa de mortalidade evitável; Comparação da prevalência de doenças crônicas; autoavaliação de saúde; fatores de risco para a saúde; e Comparação da cobertura de serviços de saúde. Na educação, foram analisados: Comparação das taxas de alfabetização e conclusão escolar; Comparação do investimento em educação; e Resultados do Programa Internacional de Avaliação de Alunos. No desenvolvimento social, foram analisadas: Comparação das taxas de pobreza; Comparação da desigualdade de renda; Taxa de desemprego; e Taxa de inclusão social. Ao final, foi elaborada uma síntese comparativa entre Brasil e Portugal e a média da OCDE. Fez-se uso de métodos estatísticos, como média, mediana e desvio padrão, quando apropriado, para identificar diferenças significativas entre os países.

## RESULTADOS

Os resultados estão organizados conforme o estado de saúde, educação e desenvolvimento social no Brasil e em Portugal.

### Estado de saúde

#### Comparação da mortalidade por causa, materna e infantil

A mortalidade infantil e a mortalidade materna são importantes indicadores da qualidade e eficácia dos sistemas de saúde, bem como do nível de vulnerabilidade social dos povos. Nesse quesito, Portugal apresenta melhores resultados que o Brasil e a média da OCDE^(16,17)^.

Portugal também apresenta melhores resultados nas taxas de mortalidade por causas relacionadas ao sistema circulatório e respiratório, totalizando 222 (por 100 mil) e 82 (por 100 mil), respectivamente, enquanto que o Brasil alcançou valores de 340 (por 100 mil) na mortalidade relacionada ao sistema circulatório e 152 (por 100 mil) na mortalidade relacionada ao sistema respiratório. Portugal apresenta pior desempenho na mortalidade relacionada a neoplasias, totalizando 211 (por 100 mil), enquanto que o Brasil registrou 178 (por 100 mil), alcançando taxas inferiores à média da OCDE^(16)^.

#### Comparação da expectativa de vida e taxa de mortalidade evitável

A expectativa de vida ao nascer é um indicador fundamental para avaliar a qualidade de vida e o acesso aos cuidados de saúde. Portugal se destaca positivamente nesse aspecto, com uma expectativa de vida significativamente maior do que a média da OCDE. Em 2021-2022, a expectativa de vida em Portugal foi de 81,5 anos, enquanto que a média da OCDE foi de 80,3 anos. Já no Brasil, a expectativa de vida foi de 74,0 anos, ainda abaixo da média da OCDE^(16)^.

A taxa de mortalidade evitável reflete a eficácia dos sistemas de saúde na prevenção de mortes que poderiam ser evitadas com cuidados de saúde adequados. Portugal apresenta uma taxa de mortalidade evitável mais baixa do que a média da OCDE, sugerindo um sistema de saúde mais eficiente. No entanto, o Brasil enfrenta desafios nesse aspecto, com uma taxa de mortalidade evitável mais alta do que a média da OCDE^(16)^.

#### Comparação da prevalência de doenças crônicas

A prevalência de doenças crônicas é outro indicador importante de saúde. No Brasil, 8,8% da população adulta é afetada pelo diabetes, enquanto que, em Portugal, esse número é de 9,1%. Ambos os valores estão acima da média da OCDE, que é de 7,0%. Isso indica a necessidade de políticas de prevenção e controle mais eficazes em ambos os países^(18)^.

A proporção de subnotificação do diabetes mellitus no Brasil foi de 42,5%, chegando a 72,8% na região Norte^(19)^. Em Portugal, considerando a composição da taxa de prevalência do diabetes, em 56% dos indivíduos, esta já havia sido diagnosticada, e em 44%, ainda não tinha sido^(20)^.

#### Autoavaliação de saúde

A autoavaliação de saúde é um indicador subjetivo em que os indivíduos avaliam sua própria saúde. Em Portugal, 77,3% da população com 15 anos ou mais classificam sua saúde como boa ou muito boa, enquanto que, no Brasil, esse número é de 65,7%. Ambos os países ficam abaixo da média da OCDE, que é de 70,2%. Essa avaliação subjetiva pode ser influenciada por diversos fatores, incluindo acesso a cuidados de saúde e qualidade de vida^(1)^.

#### Fatores de risco para a saúde

Analisando os fatores de risco para a saúde, vemos que Portugal apresenta um maior porcentual de fumantes diários em comparação com Brasil. Assim, 14,2% da população portuguesa com 15 anos ou mais são fumantes diários, enquanto que, no Brasil, esse número é de 9,1%. A média da OCDE é de 15,9%. Quanto ao consumo de álcool, Portugal tem uma taxa mais alta, com 10,4 litros consumidos *per capita* com mais de 15 anos, em comparação com 9,8 litros, no Brasil, e a média da OCDE de 8,6 litros. Já a obesidade afeta 17% da população com 15 anos ou mais em Portugal e 22% no Brasil, ambas acima da média da OCDE de 18%^(16)^.

#### Comparação da cobertura de serviços de saúde

Em relação à cobertura de serviços de saúde, Portugal apresenta um alto porcentual de população coberta pelo conjunto básico de serviços, com 95,1% da população se beneficiando desse acesso. No Brasil, esse número é de 76,08%. Portugal está acima da média da OCDE, que é de 80,2%, enquanto que Brasil está abaixo. A satisfação da população com a disponibilidade de cuidados de saúde de qualidade também é mais alta em Portugal, com 73,8% da população satisfeita, enquanto que, no Brasil, esse número é de 57,4%. A média da OCDE para a satisfação da população é de 70,8%^(1,21)^.

### Educação

#### Comparação das taxas de alfabetização e conclusão escolar

Na dimensão da educação, começamos comparando as taxas de alfabetização e conclusão escolar. Portugal apresenta uma taxa de alfabetização mais alta do que o Brasil e a média da OCDE. Além disso, 96,3% da população em Portugal é alfabetizada, enquanto que, no Brasil, esse número é de 93,2%. A média da OCDE é de 98,1%. Em relação à conclusão escolar, Portugal também se destaca, com 52,7% da população completando o ensino secundário, enquanto que, no Brasil, esse número é de 47,9%. A média da OCDE é de 84,3%^(22)^.

#### Comparação do investimento em educação

O investimento em educação é fundamental para garantir a qualidade e a acessibilidade do sistema educacional. Em Portugal, o investimento por aluno é de US$9.100,00 por ano, enquanto que, no Brasil, esse número é de US$3.600,00 por ano. Ambos os valores estão abaixo da média da OCDE, que é de US$11.200,00. Essa disparidade no investimento pode explicar, em parte, as diferenças observadas na qualidade da educação entre os dois países^(2)^.

#### Resultados do Programa Internacional de Avaliação de Alunos

O PISA avalia o desempenho dos estudantes em diferentes áreas, incluindo matemática, leitura e ciências. Portugal tem alcançado resultados acima da média da OCDE em algumas áreas do PISA, enquanto o Brasil fica abaixo da média. Isso sugere uma maior qualidade na educação portuguesa em comparação com a brasileira.

O Brasil investiu, do ensino fundamental ao nível superior em 2020, US$ 4.306,00 por estudante, o equivalente a aproximadamente R$ 21.500,00, sendo que os países da OCDE investiram, em média, US$ 11.560,00, ou R$ 57.800,00, segundo relatório da OCDE^(2)^. Consequentemente, apresenta resultados acadêmicos insatisfatórios em avaliações internacionais. No PISA, o Brasil tem desempenho abaixo da média nos grupos de matemática (379, -5 que em 2018), leitura (410, -3 que em 2018) e ciências (403, -1 que em 2018). Portugal obteve um desempenho de 472 pontos em matemática (-20.6 que em 2018), 477 em leitura (-15.2 que em 2018) e 485 em ciências (-7.3 que em 2018), sendo a média da OCDE, respectivamente, 472, 477 e 485, ficando no grupo dos países não estatisticamente diferentes da média da OCDE^(23)^. Portugal, por sua vez, investiu US$12.104,00 por estudante em educação em 2020^(22)^.

De um modo geral, os resultados do PISA 2022 decresceram devido ao impacto da COVID-19. A média da OCDE caiu quase 14 pontos em literacia matemática e cerca de 10 em leitura, em comparação com o PISA 2018. No entanto, no caso do Brasil, o fechamento das escolas não mostra diferença significativa nos resultados^(24)^.

### Desenvolvimento social

#### Comparação das taxas de pobreza

Na dimensão do desenvolvimento social, a comparação das taxas de pobreza revela diferenças significativas entre Portugal e Brasil. Portugal apresenta uma taxa de pobreza mais baixa do que o Brasil e está próxima da média da OCDE. Em Portugal, 13,4% da população vive abaixo da linha de pobreza, enquanto que, no Brasil, esse número é de 21,4%. A média da OCDE é de 11,7%^(25)^.

#### Comparação da desigualdade de renda

A desigualdade de renda, medida pelo coeficiente de Gini, é mais baixa em Portugal do que no Brasil. Portugal se aproxima da média da OCDE em termos de desigualdade de renda, enquanto que o Brasil tem uma desigualdade de renda significativamente mais alta do que a média da OCDE^(26)^.

#### Taxa de desemprego

As taxas de desemprego também diferem entre os dois países. Portugal tem uma taxa de desemprego mais baixa em comparação com o Brasil. Em Portugal, 6,6% da população está desempregada, enquanto que, no Brasil, esse número é de 14%^(26)^.

#### Taxa de inclusão social

Portugal e Brasil enfrentam desafios em relação à inclusão social. Ambos os países têm taxas de inclusão social abaixo da média da OCDE. Em Portugal, 17,2% da população enfrenta exclusão social, enquanto que, no Brasil, esse número é de 24,8%. A média da OCDE é de 14,3%^(25,26)^.

### Síntese comparativa entre Brasil e Portugal e a média da Organização para a Cooperação e Desenvolvimento Econômico

A síntese comparativa dos indicadores de Brasil e Portugal com a média da OCDE está representada na Tabela 1.

**Tabela 1:** Indicadores de Brasil e Portugal e a média da Organização para a Cooperação e Desenvolvimento Econômico

	Ano	Brasil	Portugal	Média OCDE
Estado de saúde				
Expectativa de vida (anos)	2022	74,0	81,5	80,3
Mortalidade evitável (por 100 mil)	2021	257	114	158
Prevalência de diabetes (%)	2021	8,8%	9,1%	7,0%
Mortalidade infantil (por 100 mil)	2021	12,5	2,4	4,0
Mortalidade materna (por 100 mil)	2020	72,2	11,8	10,9
Taxa de mortalidade por causa relacionada ao sistema circulatório (por 100 mil)	2021	340	222	286
Taxa de mortalidade por causa relacionada a neoplasias (por 100 mil)	2021	178	211	202
Taxa de mortalidade por causa relacionada ao sistema respiratório (por 100 mil)	2021	152	82	67
Cobertura de serviços essenciais - Atenção Básica (%)	2020	76,08	95,10	80,20
População >15 anos que fuma diariamente (%)	2021	9,1	14,2	15,9
População >15 anos que consome álcool diariamente (%)	2021	9,8	10,4	8,6
Taxas autorreferidas de sobrepeso e obesidade entre adultos (%)	2021	22	17	18
Autoavaliação de saúde	2021	65,7	77,3	70,2
Educação				
Taxa de alfabetização (%)	2022	93,2%	96,3%	98,1%
Taxa de conclusão escolar do ensino secundário (%)	2022	47,9%	52,7%	84,3%
Desempenho no PISA na categoria matemática (pontos)	2022	379	472	472
Desempenho no PISA na categoria leitura (pontos)	2022	410	477	477
Desempenho no PISA na categoria ciências (pontos)	2022	403	485	485
Desenvolvimento social				
Taxa de pobreza (%)	2022	21,4%	13,4%	11,7%
Desigualdade de renda (pontos)	2020	48,9	34,7	33,13
Taxa de desemprego (%)	2021	14%	6,6%	6,1
Taxa de exclusão social (%)	2022	24,8%	17,2%	14,3%

*Fonte: PISA (2022); OCDE (2023); e-gestor (2021).*

*OCDE - Organização para Cooperação e Desenvolvimento Econômico; PISA - Programa Internacional de Avaliação de Alunos.*

## DISCUSSÃO

Os resultados revelam diferenças significativas entre as situações de saúde, educação e desenvolvimento social no Brasil e em Portugal, bem como em comparação com a média da OCDE. A dimensão continental do Brasil, em comparação com Portugal, pode ser um dos fatores que dificultam a implementação de políticas públicas mais assertivas e equitativas. Agora, discutiremos esses resultados à luz dos ODS da ONU, explorando como esses indicadores se relacionam com os ODS e as implicações para as políticas públicas.

### Saúde e Objetivo de Desenvolvimento Sustentável 3 - Saúde e Bem-Estar

O ODS 3 da ONU visa assegurar uma vida saudável e promover o bem-estar para todos, em todas as idades. A análise dos indicadores de saúde nos dois países mostra que Portugal apresenta melhor desempenho em relação a esse objetivo do que o Brasil.

Portugal supera o Brasil em expectativa de vida, taxa de mortalidade evitável e autoavaliação de saúde. Isso sugere que as políticas de saúde em Portugal têm sido mais eficazes na promoção da saúde e na prevenção de doenças. No entanto, ambos os países enfrentam desafios relacionados às doenças crônicas e à equidade no acesso aos serviços de saúde. O envelhecimento da população portuguesa coloca desafios específicos, especialmente no campo da saúde, o que requer uma abordagem integradora em termos de políticas públicas^(27)^.

A literatura sinaliza três prioridades para o sistema nacional de saúde português, que são a melhoria da qualidade de acesso, da motivação dos profissionais de saúde para aumentar a sua produtividade e do aporte de recursos financeiros para custeio e investimento das ações em saúde^(28)^.

O sistema de saúde no Brasil se destaca positivamente por apresentar uma cobertura universal. No entanto, enfrenta desafios com a qualidade e a eficiência dos serviços, além de disparidades regionais significativas. Já o sistema de saúde português é bem avaliado em termos de acesso e qualidade. Para o Brasil, alcançar o ODS 3 exigirá um aumento significativo nos investimentos em saúde, melhoria na qualidade dos serviços e a promoção de estratégias de prevenção de doenças. A digitalização da saúde pode ser um catalisador para transformações nesse sentido, melhorando o acesso e a eficácia dos serviços de saúde, pois o acesso à saúde abrange a presença de recursos e serviços, bem como a aceitabilidade, que funciona como um impulso para a escolha desses serviços, pois facilita a realização de metas, ou seja, a resposta eficaz às necessidades, englobando a facilidade de acesso^(11,29-31)^.

Alinhar os objetivos e metas dos ODS com as demandas específicas do Sistema Único de Saúde (SUS) é essencial para garantir o cumprimento efetivo dos ODS, enquanto simultaneamente se fortalece a operacionalidade do SUS. Ao incorporar as metas dos ODS no planejamento e execução das políticas de saúde, cria-se um caminho viável para alcançar a universalização e a equidade em saúde^(4)^.

### Educação e Objetivo de Desenvolvimento Sustentável 4 - Educação de Qualidade

Desde 2000, o mundo registrou progressos significativos na área da educação marcados pelo estabelecimento dos seis objetivos da Educação para Todos e dos Objetivos de Desenvolvimento do Milênio. Apesar dos esforços, essas metas não foram atingidas dentro do prazo estipulado para 2015, destacando a necessidade de um comprometimento contínuo para concluir a agenda inacabada e, portanto, cá estamos com a Agenda 2030 para o Desenvolvimento Sustentável “Assegurar a educação inclusiva e equitativa de qualidade, e promover oportunidades de aprendizagem ao longo da vida para todos”^(5)^.

O ODS 4 da ONU busca garantir uma educação inclusiva, equitativa e de qualidade, promovendo oportunidades de aprendizado ao longo da vida para todos. A análise dos indicadores educacionais revela que Portugal está mais alinhado com esse objetivo do que o Brasil.

Ao reconhecer a relevância da educação, a Agenda 2030 para o Desenvolvimento Sustentável destaca a educação como um objetivo independente (ODS 4), incorporando também metas educacionais em vários outros ODS, especialmente aqueles relacionados à saúde, crescimento econômico, emprego, produção e consumo sustentável, e mudança climática. A educação, de fato, tem o potencial de impulsionar o avanço para a consecução de todos os ODS e, portanto, deve integrar as estratégias para alcançá-los. A nova abordagem educacional delineada no ODS 4 é abrangente, holística, ambiciosa e global, guiada por uma visão transformadora da educação que impacta positivamente a vida de indivíduos, comunidades e sociedades, assegurando que ninguém seja deixado para trás^(5)^.

Portugal supera o Brasil em taxas de alfabetização, conclusão escolar e desempenho no PISA. Isso demonstra que as políticas educacionais em Portugal têm sido mais eficazes na promoção de uma educação de qualidade e acessível. O investimento em educação e as reformas implementadas têm sido fundamentais para esse progresso.

No caso do Brasil, alcançar o ODS 4 exigirá um aumento significativo nos investimentos em educação, bem como esforços para melhorar a qualidade do ensino e reduzir as desigualdades educacionais. A expansão do acesso ao ensino superior também deve ser acompanhada pela promoção de oportunidades reais de emprego, alinhando-se com o ODS 8.

Para haver possibilidade de mudanças e alcance desse objetivo, os governos devem assumir a responsabilidade primordial de garantir o direito à educação, desempenhando um papel central como zeladores do gerenciamento e financiamento eficiente, equitativo e eficaz da educação pública e mantendo a liderança política no setor educacional. Cabe a eles orientar o processo de contextualização e implementação dos objetivos e metas estabelecidos pela educação, mas cabe à sociedade ficar vigilante^(5,31)^.

### Desenvolvimento social e Objetivo de Desenvolvimento Sustentável 1 - Erradicação da Pobreza e Objetivo de Desenvolvimento Sustentável 10 - Redução das Desigualdades

Os ODS 1 e 10 da ONU preconizam a erradicação da pobreza e a redução das desigualdades. A análise dos indicadores de desenvolvimento social revela diferenças significativas entre Brasil e Portugal.

Portugal possui uma taxa de pobreza mais baixa e menor desigualdade de renda que o Brasil. Isso sugere que as políticas sociais em Portugal têm sido mais eficazes na promoção da inclusão social e na redução das disparidades econômicas. No entanto, ambos os países enfrentam desafios em relação à inclusão social, com taxas abaixo da média da OCDE.

A desigualdade marcante no Brasil, alinhada ao ODS 10, questiona não apenas políticas de redistribuição de renda, mas a própria estrutura social. Como as intervenções podem ir além de mitigar, buscando eliminar as raízes estruturais das desigualdades? Portugal, enfrentando desafios mesmo com desigualdades menores, levanta questões sobre a natureza dessas desigualdades. Como políticas podem ser adaptadas para lidar com desafios específicos, respeitando a singularidade do contexto português?

No Brasil, alcançar os ODS 1 e 10 exigirá a implementação de políticas públicas voltadas para a redução da pobreza e da desigualdade, progressivamente alcançando e sustentando o crescimento da renda dos 40% da população mais pobres a uma taxa maior que a renda média dos 10% mais ricos, bem como a promoção de oportunidades econômicas para todos. A inclusão social deve ser priorizada, com ações que atendam aos grupos mais vulneráveis^(10-12,30,31)^.

O enfrentamento dos problemas identificados é fortemente dependente de macro-políticas que tenham continuidade e identidade, com valores de igualdade, solidariedade e respeito humano.

### Comparação com a média da Organização para a Cooperação e Desenvolvimento Econômico e aspectos éticos

A comparação com a média da OCDE mostra que tanto Portugal quanto Brasil têm áreas que estão abaixo desse padrão internacional. Isso destaca a complexidade dos desafios enfrentados por ambos os países e a necessidade de ações para melhorar seus sistemas de saúde, educação e desenvolvimento social.

Além disso, é fundamental considerar os aspectos éticos envolvidos, ao analisar indicadores relacionados à saúde, educação e desenvolvimento social. As políticas públicas devem ser guiadas por princípios éticos que promovam a igualdade de acesso, a justiça social e o respeito aos direitos humanos. A coleta e o uso de dados também devem ser conduzidos de maneira ética, garantindo a privacidade e a confidencialidade das informações dos cidadãos.

### Limitações do estudo

Por fim, algumas limitações deste estudo incluem as diferenças de escala e população, o contexto histórico e cultural, a diversidade interna, os sistemas políticos e administrativos distintos, as desigualdades sociais e econômicas, e a definição e mensuração de indicadores.

### Contribuições para a área da enfermagem, saúde ou política pública

Este estudo enriquece o debate sobre políticas públicas na área da saúde, destacando a importância de uma abordagem multifacetada, que considera não apenas indicadores de saúde, mas também fatores educacionais e de desenvolvimento social. As descobertas apontam para a necessidade de políticas que promovam a equidade e a eficiência, incentivando o Brasil e Portugal a se inspirarem mutuamente nas áreas onde cada um se sobressai. Ao sublinhar o papel central da saúde no desenvolvimento sustentável, o estudo convida formuladores de políticas a considerarem investimentos estratégicos de longo prazo que abordem não só as necessidades imediatas, mas que também estabeleçam as bases para sociedades mais saudáveis e resilientes.

## CONSIDERAÇÕES FINAIS

Os indicadores de saúde, educação e desenvolvimento social do Brasil e Portugal, analisados e relacionados com a média da OCDE, mostram uma visão das disparidades entre os dois países. Portugal se destaca positivamente em várias áreas, incluindo expectativa de vida, qualidade da educação e taxas de pobreza. No entanto, ambos os países enfrentam desafios, como a alta prevalência de doenças crônicas e a necessidade de investimentos em educação.

A análise comparativa realizada mostra que ambos os países têm aspectos nos quais se alinham ou divergem da média da OCDE, com Portugal sendo geralmente mais próximo dos padrões da OCDE do que o Brasil. A análise ressalta, também, a complexidade e a variedade dos desafios enfrentados por Brasil e Portugal, ilustrando como fatores históricos, econômicos e políticos influenciam o desempenho em saúde, educação e desenvolvimento social. As tendências indicam áreas de progresso e oportunidades para reformas e investimentos futuros que podem orientar políticas públicas nos campos da saúde, educação e desenvolvimento social.

Ao destacar as diferenças e semelhanças entre Brasil e Portugal em relação às médias da OCDE em saúde, educação e desenvolvimento social, este estudo oferece uma visão abrangente dos desafios a serem enfrentados por ambos os países, assim como áreas de destaque que podem servir de modelo para outras nações.

É imperativo que tanto o Brasil quanto Portugal mantenham um compromisso contínuo com melhorias no sistema de saúde. Uma ênfase especial na integração da saúde digital e na distribuição equitativa de recursos se apresenta como uma necessidade premente. Além disso, a adoção de políticas alinhadas com os ODS da ONU é crucial para assegurar avanços consistentes em direção a sistemas de saúde mais eficientes e equitativos.

O desafio futuro reside na persistência dos investimentos, reconhecendo que a saúde é um pilar fundamental para o desenvolvimento sustentável. O caminho para sistemas de saúde mais robustos exige não apenas resolução de desafios imediatos, mas também uma visão estratégica que antecipe e responda prontamente às futuras demandas da sociedade.
